# Late HIV Diagnosis: Proposed Common Definitions and Associations With Short-Term Mortality

**DOI:** 10.1097/MD.0000000000001511

**Published:** 2015-09-11

**Authors:** Hongbo Jiang, Nianhua Xie, Jianhua Liu, Zhixia Zhang, Li Liu, Zhongzhao Yao, Xia Wang, Shaofa Nie

**Affiliations:** From the Department of Epidemiology and Biostatistics, School of Public Health, Tongji Medical College, Huazhong University of Science and Technology, Wuhan, China (HJ, JL, ZZ, LL, SN); and Wuhan Center for Disease Control and Prevention, Wuhan, China (NX, ZY, XW).

## Abstract

The aim of this study was to present a definition of late presentation according to different time periods between initial diagnosis of human immunodeficiency virus (HIV) infection and acquired immune deficiency syndrome (AIDS) diagnosis which would reliably identify individuals with high risk of mortality within 1 year of diagnosis, and could be used as a suggested common definition.

Data of individuals diagnosed from 1994 to February 2012 in Wuhan, China were extracted retrospectively from the national HIV surveillance system. Four time periods (1, 3, 6, and 12 months) combined with the European consensus definition of advanced HIV disease (AHD) were compared. The predictive ability of each definition for identifying an individual who died within 1 year after HIV diagnosis was assessed.

A total of 980 patients were included, of whom 289 (29.49%), 324 (33.06%), 353 (36.02%), and 387 (39.49%) were defined as AHD according to the definition of a CD4 count <200 cells/μL or AIDS-defining event (ADE) within 1, 3, 6, and 12 months of HIV diagnosis, respectively. One hundred twenty-seven (12.96%) patients died within 1 year of diagnosis. The highest Youden's index and largest area under the curve were presented in time period within 3 months. Time period within 1 month presented the highest consistency rate, positive likelihood ratio, and kappa value. Longer time periods increased the sensitivity but decreased the specificity.

Given the European consensus definitions and the current results, we suggested that AHD could be defined as “a first-reported CD4 count <200 cells/μL or an ADE within 1 month after HIV diagnosis.” “Late presentation” could be defined as “a first-reported CD4 count <350 cells/μL or an ADE within 1 month after HIV diagnosis.”

## INTRODUCTION

Since the antiretroviral therapy (ART) was available, the morbidity and mortality of people living with human immunodeficiency virus (HIV) infection and acquired immune deficiency syndrome (AIDS) have reduced dramatically, which transformed HIV/AIDS into a chronic manageable disease rather than an inevitably progressive and terminal illness.^1,2^ However, a significant proportion of individuals cannot take full advantage of the treatment, in particular individuals who are unaware of their positive HIV serostatus, and those who begin therapy in late course of disease.^3,4^ A late diagnosis of HIV infection is detrimental to health for individuals in terms of increased morbidity and mortality as well as a higher risk of clinical events and hospitalizations, for public health in terms of increased potential for transmission from individuals unaware of their HIV status and individuals who are not in care and with uncontrolled viral load, for healthcare systems in terms of increased resource burden and costs.^5–8^

Over 20 different criteria have been used to define late presentation, which generally include CD4 cell count and/or AIDS-defining diseases.^8,9^ The lack of a consistent definition will have an impact on the apparent prevalence of late presentation, and has also hampered attempts to assess temporal trends after targeted interventions, to make cross-country or regional comparisons, and to identify risk factors for late presentation.^6,10^ In response to the heterogeneity of criteria to define late presentation, the European Late Presenter Group (ELPG) proposed 2 consensus definitions of late HIV diagnosis that “late presentation” was defined as the presence of an AIDS condition or CD4 cell count <350 cells/L at presentation for care, and “advanced HIV disease” (AHD) or as the presence of either an AIDS condition or a CD4 cell count <200 cells/L at presentation.^10–12^ Though the consensus definitions have been adopted in the literature, the inconsistency of definitions still exists because of varied short-time periods between initial diagnosis of HIV infection and AIDS diagnosis ranging from 1 to 12 months used to define late HIV diagnosis.^5,6^

So there is a clear and desirable need for a definition which specifies the short-time period between initial diagnosis of HIV infection and AIDS diagnosis. Given the current situation, we used HIV surveillance data in Wuhan, China to investigate the use of several common short-time periods in definitions of AHD with the aim of providing suggestion for a definition that can reliably identify a high proportion of individuals who will die within 1 year after HIV diagnosis.

## METHODS

### Participants

All individuals with HIV positive in China are reported to the Center for Disease Control and Prevention (CDC) through the national HIV surveillance system, which was then upgraded to the China Information System for Disease Control and Prevention (CISDCP) in 2003.^13^ As the capital city in the Hubei province in central China, Wuhan has a population of 10.22 million people and developed the web-based reporting system for conventional infectious diseases in September 2003 and then upgraded the system to a special reporting system for AIDS and tuberculosis in 2005. The information for individuals with HIV positive was sent to the Wuhan CDC via email before 2003 and then manually entered into the system after the reporting system was developed.^14^ Data from 1994 to February 2012, including demographic characteristics, HIV diagnosis, AIDS diagnosis, and CD4 cell count were extracted from the database, which were described in our previous study.^14^ The extracted raw data were first edited by the logical check. Individuals who were reported by the Wuhan CDC, confirmed to be HIV-infected by a positive western blot, Wuhan residents, and ages older than 13 years at diagnosis were included in our study. A total of 980 cases met the inclusion criteria.

### Data Analysis

Descriptive results for quantitative variables are expressed as medians and interquartile ranges (IQRs). We define AHD as a first-reported CD4 count <200 cells/μL or presenting with an AIDS-defining event (ADE), regardless of the CD4 cell count, according to a consensus definition proposed by ELPG.^10–12^ The different time periods (within 1 month, within 3 months, within 6 months, within 12 months), which were commonly presented in the literature,^6,15^ were used to specified time between a first-reported CD4 count <200 cells/μL or ADE and HIV diagnosis in the definition of AHD. The predictive ability (described by the sensitivity, specificity, Youden's index, positive likelihood ratio [+LR], negative likelihood ratio [–LR], consistency rate, kappa value, positive predictive value [PPV], negative predictive value [NPV], and receiver operating characteristic [ROC] curve) of each definition for identifying individuals who died in the first 12 months after HIV diagnosis (often referred to as short-term mortality) was assessed. The area under the curve (AUC) and its 95% confidence interval (CI) was used to compare the predictive ability of each definition. Data analyses were performed using SPSS (version 12.0; SPSS Inc., Chicago, IL).

### Ethics Statement

The institutional review board of Tongji Medical College of Huazhong University of Science and Technology approved the study. Written consent was obtained from the Wuhan CDC for approving the use of data in this study. Patient consent was waived in the current retrospective study because of no anticipated risks for the participants.

## RESULTS

A total of 980 cases diagnosed with HIV positive were included. Men were diagnosed more frequently than women (sex ratio: 4.54). The median age at HIV diagnosis was 36 years, ranging from 17 to 85 years (IQR: 27–47 years old). Sexual contact was the main route of HIV transmission: 40.71% of cases involved men who had sex with men, and 41.12% involved heterosexual contact. Injection drug users, individuals infected through blood transfusion or blood products, and other unknown reasons accounted for 2.54%, 6.94%, and 1.63%, respectively. Of note, CD4 cell counts were unavailable for 12.35% patients.

Of the 980 cases, 289 (29.49%) were defined as AHD according to the definition of “a first-reported CD4 count <200 cells/μL or an ADE within 1 month of HIV diagnosis.” Among them, 194 cases presented with a first-reported CD4 count <200 cells/μL and the other 95 cases presented with an ADE. The numbers of cases defined as AHD according to time period between CD4 count <200 cells/μL or an ADE and of HIV diagnosis were 324 (33.06%) within 3 months, 353 (36.02%) within 6 months, 387 (39.49%) within 12 months (Table 1).

**TABLE 1:**
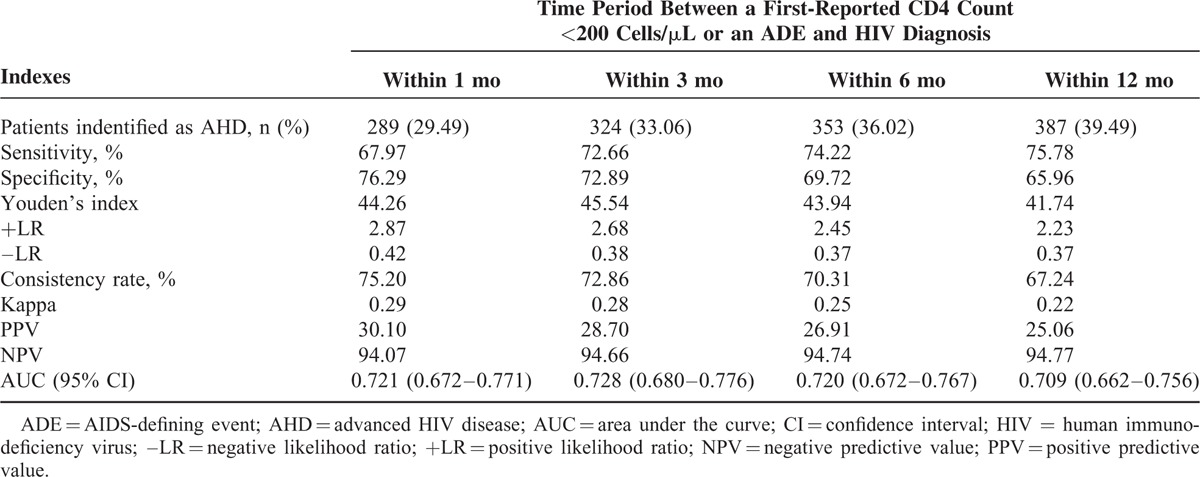
Predictive Ability of Several Commonly Used Definitions of Advanced HIV Disease for Short-Term Mortality

**FIGURE 1:**
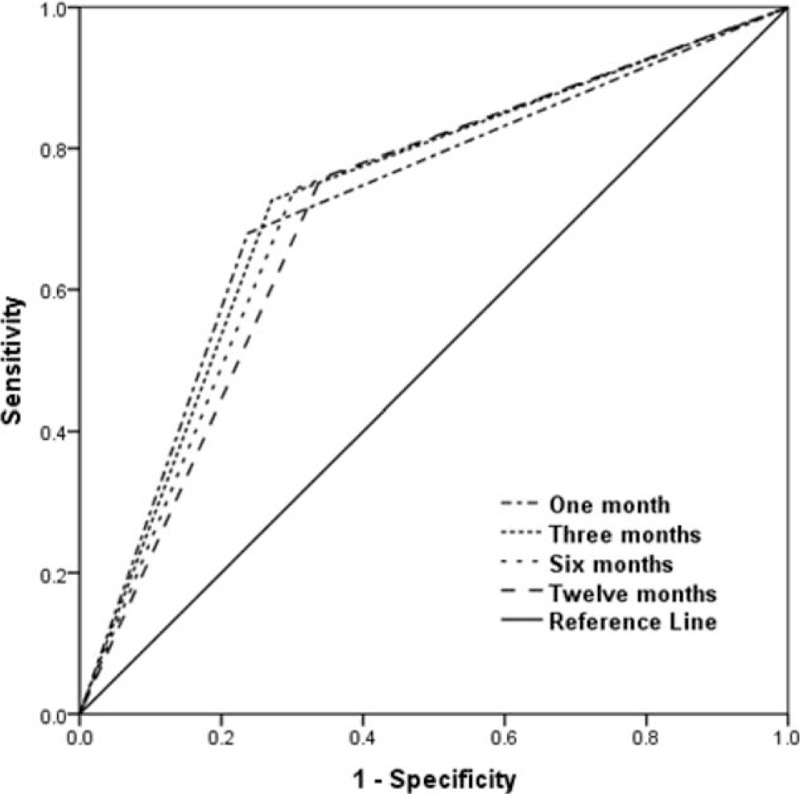
Receiver operating characteristic curve of each definition for identifying individuals who died in the first year after human immunodeficiency virus diagnosis.

A total of 127 (12.96%) died within 1 year of diagnosis. Definitions based on longer time periods which classified patients as AHD, generally resulted in increased sensitivity and NPV than definitions based on shorter time periods, but reduced specificity and PPV. Time period within 1 month presented the highest consistency rate, +LR, and kappa value. The highest Youden's index was presented in time period within 3 months. As for results of ROC curve, time period within 3 months had the largest AUC. However, the difference of AUC in the 4 time periods was not statistically significant according to the 95% CI (Table 1; Figure 1).

## DISCUSSION

A consensus definition of late presentation would be beneficial to allow the identification of risk factors. Trends of late presentation over time and changes in rates of late presentation after interventions for earlier diagnosis could be effectively monitored in a common way.^9,11^

The definitions based on time periods had an impact on the prevalence of AHD. The definition with a time period of 12 months identifies the most individuals as presenting with AHD, and had the highest sensitivity to identify individuals who die within 12 months after HIV diagnosis. But it is worth noting that high sensitivity is often achieved at the expense of high specificity.^11^ Moreover, the longer time periods would not facilitate to early identify individuals at higher risk of mortality within 12 months after HIV diagnosis. Since timely ART initiation and adherence to treatment might have an influence on the occurrence of ADEs over the first year in care, longer time periods might provide imprecise estimates of late diagnosis and presentation by incorporating other care utilization processes. Thus, a time period of 3 months was suggested according to a balance between missing data with a shorter window (eg, 1 month) and misclassification due to other healthcare processes with a longer window (eg, 12 months) in a previous study.^5^ The highest Youden's index and largest AUC were presented in time period with 3 months in the present study. Time period within 1 month presented the highest consistency rate, +LR, and kappa value. Given our results, we suggested that shorter time periods were more reliable to identify an individual at high risk of mortality within 12 months after HIV diagnosis, to be more specified, a 1-month time period could be possibly used in the consensus definition of late HIV diagnosis, which was consistent with the suggestion of the UK Collaborative HIV Cohort (UK CHIC) Steering Committee.^11^

In our study, we have focused on mortality within 12 months after HIV diagnosis which was commonly used in the existed literature.^16–18^ Individuals with late presentation experienced a particularly high rate of mortality within 12 months after HIV diagnosis. A previous study showed that the overall mortality rates of late presenters and nonlate presenters were 12.8 and 1.7/100 person-years, respectively, and these differences were greatest in the first year after HIV diagnosis (24.4 vs 0.3 per 100 person-years).^19^

It is estimated that 437,000 people were living with HIV/AIDS in China by the end of 2013 according to the China AIDS response progress report in 2014. The epidemic remains serious and complex, although progress in HIV prevention and control has been made.^20^ However, few evidences for late presentation in mainland China have been presented. One cross-sectional study conducted 10 provinces of mainland China from 2009 to 2010 indicated that 72.02% of the patients had a CD4 count ≤200 cells/μL at HIV diagnosis, whereas patients with a CD4 count <100 cells/μL accounted for 53.98%, and patients with a CD4 count ≥350 cells/μL only accounted for 8.75%.^21^ Another study conducted in Liuzhou city showed that 72.6% of the 899 participants had a late diagnosis which was defined as either a concurrent AIDS diagnosis at the time of HIV diagnosis or developing AIDS within 1 year after HIV diagnosis.^22^ More attention should be paid to the issue of late presentation due to the sizeable population of late presentation in China. Additionally, no study was conducted to qualify the predict ability of definitions with different time periods for identifying individuals who died in the first 1 year after HIV diagnosis. It would be desirable to access the validation of our findings in other studies.

Besides, it should be noted that the data used in this study were people with residence in Wuhan which may restrict the generalizability of findings to other settings, although Wuhan has a population of 10.22 million. And last but not the least, we hope more experts in this field could be interested in the exploration of the time periods, and make efforts for the consensus definition of late presentation together.

## CONCLUSIONS

Given the European consensus definitions and the current results, the common definitions of late HIV diagnosis are suggested that AHD could be defined as “a first-reported CD4 count <200 cells/μL or an ADE within 1 month after HIV diagnosis.” “Late presentation” could be defined as “a first-reported CD4 count <350 cells/μL or an ADE within 1 month after HIV diagnosis.”
